# A qualitative comparison of primary care clinicians’ and their patients’ perspectives on achieving depression care: implications for improving outcomes

**DOI:** 10.1186/1471-2296-15-13

**Published:** 2014-01-15

**Authors:** Robert D Keeley, David R West, Brandon Tutt, Paul A Nutting

**Affiliations:** 1 Department of Family Medicine, University of Colorado, Denver, Mail Stop F-496, Academic Office 1, 12631 E. 17th Ave, Aurora, CO 80045, USA; 2 Denver Health and Hospital, 301 West 6th Avenue, Denver, CO 80204, USA; 3 Center for Research Strategies, 225 E. 16th Ave, Denver, CO 80203, USA

**Keywords:** Depression, Patient-centered, Qualitative research

## Abstract

**Background:**

Improving the patient experience of primary care is a stated focus of efforts to transform primary care practices into “Patient-centered Medical Homes” (PCMH) in the United States, yet understanding and promoting what defines a positive experience from the patient’s perspective has been de-emphasized relative to the development of technological and communication infrastructure at the PCMH. The objective of this qualitative study was to compare primary care clinicians’ and their patients’ perceptions of the patients’ experiences, expectations and preferences as they try to achieve care for depression.

**Methods:**

We interviewed 6 primary care clinicians along with 30 of their patients with a history of depressive disorder attending 4 small to medium-sized primary care practices from rural and urban settings.

**Results:**

Three processes on the way to satisfactory depression care emerged: 1. a journey, often from fractured to connected care; 2. a search for a personal understanding of their depression; 3. creation of unique therapeutic spaces for treating current depression and preventing future episodes. Relative to patients’ observations regarding stigma’s effects on accepting a depression diagnosis and seeking treatment, clinicians tended to underestimate the presence and effects of stigma. Patients preferred clinicians who were empathetic listeners, while clinicians worried that discussing depression could open “Pandora’s box” of lengthy discussions and set them irrecoverably behind in their clinic schedules. Clinicians and patients agreed that somatic manifestations of mental distress impeded the patients’ ability to understand their suffering as depression. Clinicians reported supporting several treatment modalities beyond guideline-based approaches for depression, yet also displayed surface-level understanding of the often multifaceted support webs their patient described.

**Conclusions:**

Improving processes and outcomes in primary care may demand heightened ability to understand and measure the patients’ experiences, expectations and preferences as they receive primary care. Future research would investigate a potential mismatch between clinicians’ and patients’ perceptions of the effects of stigma on achieving care for depression, and on whether time spent discussing depression during the clinical visit improves outcomes. Improving care and outcomes for chronic disorders such as depression may require primary care clinicians to understand and support their patients’ unique ‘therapeutic spaces.’

## Background

The United States is attempting to improve clinical outcomes in a more cost-effective [1] and equitable manner [2,3] by disseminating a set of dimensions and services called the “Patient-Centered Medical Home” (PCMH) [4]. This focus on creating a 21^st^ century PCMH in the United States recapitulates previous efforts in this country, [5,6] and parallels ongoing efforts to improve primary care processes and outcomes in Europe and Australia [7,8]. The National Center for Quality Assurance (NCQA), which determines if primary care practices are awarded PCMH status, has defined PCMH as “a way of organizing primary care that emphasizes care coordination and communication to transform primary care into what patients want it to be” [1]. PCMH was designed by health care organizations in 2007. Subsequently, NCQA has been criticized for emphasizing health information technological and communication infrastructure as part of the accreditation process at the expense of understanding and promoting key components of the patient experience [9].

The patient experience has been described as a multidimensional, complex phenomena that is influenced by individual and environmental phenomena [10,11]. Initial assessments of practice efforts to transform to PCMH have reported minimal or no improvements in the patient experience [12-14]. Likewise, Martsolf et al. found no relationship between PCMH-concordant processes and better experience ratings. The patient experience is recognized by NCQA as a critical component of quality of care, and studying the primary care patients’ experiences, expectations and preferences regarding the care they receive may begin to clarify the ‘patient experience’ and how to measure it [15].

Depression care may provide an ideal context in which to examine new challenges for designing primary care that recognizes the central import of the patient experience [15]. Major depression is prevalent [16], costly [17,18], and often takes a chronic or recurring course in primary care settings [19]. The qualitative literature describing how depressed patients interact with health care systems spans decades and is relatively comprehensive. In the early 1990’s, interviews conducted with 50 depressed persons in the United States uncovered a range of negative interactions with the mental health system [20]. Modern primary care practice may provide better experiences for many depressed persons, yet deficits persist in appreciating the broad range of needs, expectations and preferences of patients with depression [21].

It should be noted that a definition of ‘primary care’ may not be well defined or understood. Phillips and Bazemore recently surveyed the world’s literature on “primary care”, and found that it is characterized ideally by relationships between patients and teams of providers that endure over time, offers a broad scope of practice, health care integration, and transition management, and works closely with community support services as well. Robust information systems would enable quality improvement through systematic preventive services delivery, utilization monitoring, and population health tracking [22]. This definition is similar to longer definitions of PCMH, [2,3] and it is challenging for small- to medium-sized practices to implement all these features of ideal primary care [23].

A study of patient experiences as they achieved care for mental disorders in European and Australian health care systems concluded that improving access and communication during encounters, along with tailored psychosocial interventions would enhance the patient experience [24]. Kovandzic et al. described barriers to receiving care and possible solutions for ‘hard-to-reach’ persons suffering mental distress [25]. Palmer et al. convened patient and community perspectives to inform development of an effective primary care approach to depression [26]. Kokanovic et al. have begun to reconcile lay descriptions with professional definitions of depression [27].

In order to begin building upon some of these findings, we interviewed patients diagnosed with a depressive disorder attending one of 4 small- to medium-sized primary care clinics in the United States participating in quality improvement projects around depression diagnosis and care. Our goal was to learn about the patients’ experiences, expectations and preferences as they achieved care for depression. We also planned to compare the patients’ perspectives with observations elicited from their primary care clinicians, thereby generating ideas from multiple perspectives about what to measure about the patient experience and how to improve the patient’s experience in primary care.

Our research questions were: 1. What are the experiences, expectations and preferences of primary care patients as they achieve depression care? 2. How do primary care clinicians perceive the experiences, expectations and preferences of their patients as they receive care for the depression?, and 3. How does comparing the patients’ and their clinicians’ perceptions inform what to measure about the patient experience and how to begin improving the patient experience within primary care?

## Methods

This ethnographic study was designed and executed within the general framework of a larger, mixed methods comparative effectiveness trial, Enhancing Practice, Improving Care (EPIC). EPIC randomized primary care practices in Colorado to three arms of a trial designed to compare approaches to improving quality of chronic care for diabetes or depression. EPIC provides an important next step in collaborative care research that draws upon the lessons learned from a stream of investigation beginning with the Direct Observation of Primary Care study through the National Demonstration Project [28,29]. Details of the EPIC study are presented elsewhere [30].

The embedded ethnographic study was designed to generate a better understanding of primary care patients’ experiences, expectations and preferences regarding the care for mental distress they receive from the health care system. The study used semi-structured interviews with a purposeful sample of primary care clinicians and their patients with a history of depression.

### Selection of subjects

Clinician participants in the EPIC trial were invited to participate based on our early understandings of their approach to chronic care in general. Clinicians were selected and invited sequentially, with subsequent selections based on early data and an effort to get maximum variability in clinician approaches to detection and treatment of depression. One clinician declined to participate. After interviewing five family physicians and one family nurse practitioner we felt that we had reached saturation for clinicians’ general approaches to depression care. Three clinicians were interviewed prior to interviewing patients, and 3 after at least one patient from each clinician had been interviewed.

Patient respondents were drawn from lists of clients with depression, generated by participating practices in the randomized trial of practice reorganization to improve depression care. Participating clinicians reviewed the list of patients billed for depression-related ICD-9 codes in the past 12 months and eliminated those with cognitive impairment, severe psychological co-morbidities or current suicidal ideation, or for whom the clinician had any sense that participation might be harmful. The recruitment letter stated to patients that they had been diagnosed with possible symptoms of depression over the previous year and invited them to participate in a single interview to discuss their views on depression and its treatment. The patients were asked to return an enclosed postcard if they were interested. We mailed about 200 postcards to recruit 30 patient participants.

Interviews with patients were conducted sequentially as well with early interviews affecting the number of patients to be selected from each clinician. We continued to sample, conduct interviews, and analyze the data until we had data on 30 patients, at which time we felt we had reached saturation. Patient participants were recruited and interviewed between March 2009 and June 2010. All interviews were conducted over the telephone (average length 35 minutes, range 11 minutes to 64 minutes).

All participating clinicians and patients provided informed consent. The study protocol and methods were approved by the Colorado Multi-Institutional Review Board.

### Data collection

Data collection began with semi-structured telephone interviews of clinicians. An interview guide [Additional file 1] was developed by the authors and addressed basic ideas about depression and its place in overall health, approach to detecting and diagnosing depression, ideas about referral, in practice counseling and/or use of medication, and strategy for monitoring during acute phase treatment. All clinician interviews were conducted by one family physician (Author 1) and generally lasted an hour.

We began patient interviews after three of the clinicians’ interviews had been conducted and analyzed. Semi-structured telephone interviews were conducted by one author (Author 1) and one trained clinical psychologist. An interview guide [Additional file 2] provided the general framework of the semi-structured interview and addressed the patient’s general conceptualization of depression, their broad expectations of the clinician (and the practice) regarding depression assessment and treatment, recent experience with the interviewed clinician and their practice in general, and experience with mental health professionals. As the 3 emergent themes were defined, the patient interview guide was modified to collect a narrative of the patients’ personal experiences with emotional symptoms, receipt of care over time, and how they handled depression and preventing recurrent depression over time.

All interviews were audio-recorded and transcribed. Two members of the team read all interview transcripts as soon as they were available and discussed with the team. During team meetings, interviewers received feedback on content and interviewing style, and adjustments were made to interview guides as appropriate and as new ideas emerged.

### Data analysis

The authors conducted the analysis in a series of one-hour meetings; many in real-time while data collection was still underway, and a series of four half-day meetings after all data were collected. All transcripts were imported into Atlas.ti (Berlin, Germany) for management and analysis. Data were analyzed and interpreted in several phases using an immersion/crystallization process [31] with systematic, iterative process of text interpretation and categorization to establish important patterns.

Initially, the authors read the clinician and patient interviews and created brief summaries, based on a template that generally followed the interview guide. To focus on emerging themes and enhance cross-case comparisons, we later developed a matrix of key concepts and research questions that emerged from earlier analyses. This approach drew on strategies developed by Miles and Huberman [32]. This process encouraged us to return to the original transcripts to expand information in the matrix, often inserting brief excerpts of raw text to support interpretations. Sampling continued until theoretical saturation of the data was reached.

During the analysis the group maintained sensitivity to pre-conceptions that we might have brought to the analysis. This was particularly useful as two members of the group were family physicians, both with practice experience and previous depression research. The other two members included a cultural anthropologist and a master’s level public health researcher. Differences in how we interpreted the data did emerge and were resolved by continued discussion until consensus was reached.

## Results

### The sample

Table 1 describes the participating 6 clinicians (from 4 practices) and 30 patients. Clinicians were all non-Hispanic white and relatively experienced Family Practitioners and 1 Nurse Practitioner. The patients were from predominately non-Hispanic white populations, although 2 self-identified as Hispanic. Most patients were insured and had experienced multiple depressive episodes.

**Table 1:** Description of participating primary care practices, clinicians, and patients

**Practice description**	**Primary care clinician gender/type/age (years)**	**Patient gender/age (years)**	**Insurance type/employment/marital status/other**	**Single/multiple episodes of depression/psychiatric comorbidity**
Practice A: 7-clinician practice in Denver Suburb.	Clinician a: female family physician, 54	Patient 1: F(female)/51-55	NA (not available)	Multiple
Physician-owned practice.				
Patients referred outside of practice to therapy/psychiatry.		Patient 2: F/26-30	Private insurance/unemployed/single student	Multiple, comorbid obsessive compulsive disorder
Quality Improvement (QI) Project: developing an electronic patient tracking system including web messaging to communicate with patients.		Patient 3: F/NA	Private insurance/NA/divorced	Multiple
		Patient 4: F/26-30	Private insurance/unemployed/married/pregnant	Multiple
		Patient 5: F/36-40	Private insurance/NA/widowed	Multiple
		Patient 6: F/46-50	Private insurance/employed/divorced/non-Hispanic white	Multiple
		Patient 7: F/36-40	Private insurance/employed	
		Patient 8: M (male)/51-55	Private insurance/Employed	Multiple
		Patient 9: F/61-65	Private insurance/retired/married/grandchildren	Multiple
	Clinician b: male family physician, 54	Patient 10: F/21-25	Private insurance/employed/married	Multiple
	Clinician c: female family physician, 48	Patient 11: F/26-30	NA	Single
		Patient 12: F/51-55	Private insurance/employed	Multiple
		Patient 13: F/61-65	NA	Multiple
		Patient 14: F/51-55	Private insurance/employed	Multiple/comorbid fibromyalgia
	Multiple clinicians	Patient 15: F/26-30	Public insurance/unemployed/single	Multiple
	Multiple clinicians	Patient 16 Patient 12: F/NA	NA	Multiple
	Multiple clinicians	Patient 17: M/NA	Uninsured/unemployed	Multiple
	Multiple clinicians	Patient 18: M/NA	NA	Multiple
	Multiple clinicians	Patient 19: F/66-70	Private insurance/retired	
	Multiple clinicians	Patient 20: F/26-30	Private insurance/Employed/divorced	
Practice B: 3-clinician practice in large town of 80,000 people.	Clinician d: male family physician, 38	Patient 21: F/36-40	Insured/homemaker/married/Hispanic	Multiple
Physician-owned practice.				
Patients referred outside of practice to therapy/psychiatry.				
QI project: targeted depression screening and symptom tracking of newly diagnosed patients.				
Practice C: solo practice in Denver Suburb.	Clinician e: male family physician, 45	Patient 22: F/61-65	Uninsured/unemployed/divorced	Multiple
Physician-owned practice.		Patient 23: F/71-75	Insured/homemaker/married/caregiver/ gravely ill husband	Multiple
Co-located with psychiatric office. patients referred to therapy outside of practice location.				
	
QI project: targeted depression and anxiety screening and symptom tracking of newly diagnosed patients.		Patient 24	Insured/retired/married	Multiple
		Patient 25: M/36-40	Insured/unemployed/engaged to be married	Multiple
		Patient 26: F/36-40	Insured/self-employed/married	Single
		Patient 27: F/46-50	Insured/employed/divorced	Single
Practice D: 2-clinician Federally Qualified Community Health Center.	Clinician f: female nurse practitioner, 49	Patient 28: F/26-30	Uninsured/unemployed/divorced/High school education	Multiple
Mountain town of 15,000 people.				
		Patient 29: F/66-70	Insured/ homemaker/married	Multiple
Co-located with a community mental health organization.				
QI project: improve integration with mental health center.				
		Patient 30: F/61-65	Insured/employed/divorced	Multiple

Three key themes emerging from the qualitative analyses were the (1) journey to achieving depression care, (2) search for a very personal understanding of their depression, and (3) creation of a ‘therapeutic space’ in which to receive depression care.

We classified subthemes into facilitators and barriers, and categorized them according to 3 important domains of health care: (clinician) competency, relational, and systems, [26] and added a (patient-related) ‘clinical’ domain. We compared the subthemes generated by patients to those originating from clinicians.

The clinician- and patient-level facilitators and barriers for the 3 themes are presented in Table 2, and are discussed below.

**Table 2:** Clinician and patient themes and subthemes

**Clinician themes/subthemes**	**Patient themes/subthemes**
**Theme #1 - Patient’s journey to receive depression care**	**Theme #1- Patient’s journey to achieving depression care**
*Subtheme Facilitators*	*Subtheme Facilitators*
a. Quality improvement projects (S*)	a. Supportive family members (R*)
b. Clinic staff training (S)	b. Friendly office atmosphere (R)
c. Television advertisements for antidepressant medication (S)	
*Subtheme Barriers*	*Subtheme Barriers*
a. Fragmented specialty mental health sector (S)	a. Fragmented health care system (S)
b. Fear of opening “Pandora’s box” (S, R)	b. Stigma (R)
c. Payment structure (S)	c. Rushed primary care clinicians (S)
d. Poor patient follow-up (S)	d. Clinicians appearing to lack adequate mental health expertise (C*)
	e. Perceived potential for inappropriate office staff behaviors (S)
**Theme #2 - Aiding patients to come to a personal understanding of depression**	**Theme #2 - The search for a very personal understanding of their depression**
*Subtheme Facilitators*	*Subtheme Facilitators*
a. Education (C, R)	a. Family history, contact with depressed person in past (R)
b. Screening for bipolar disorder (S, Cl*)	b. History of psychotherapy or counseling (R)
*Subtheme Barriers*	*Subtheme Barriers*
a. Somatic presentation (Cl, R)	a. Somatic presentation (Cl, R)
b. Patient’s secondary gain agenda (S, R)	
c. Physical pain (Cl)	
d. Substance abuse (Cl)	
e. Stigma (R)	
**Theme #3 – Supporting the patient’s therapeutic space**	**Theme #3 - Creation of a personalized therapeutic space**
*Subtheme Facilitators of an effective therapeutic space*	*Subtheme Facilitators of an effective therapeutic space*
a. Antidepressant medication. (Cl)	a. Antidepressant medication (Cl)
b. Counseling/Psychotherapy (Cl, R, S)	b. Counseling/Psychotherapy (Cl, R, S)
c. Primary care clinician (C, Cl, R, S)	c. Primary care clinician (C, Cl, R, S)
- Multifaceted, team approach (C, Cl, R, S)	d. Therapeutic aspects of working (R, S)
- Emphasis on follow-up compliance (Cl, R)	e. Values
- Empathetic, active listening (R)	f. Nature and Family (R, S)
d. Physical activity (Cl)	g. Faith (R, S)
e. Social support (R)	h. Positive memories (Cl, R)
f. Depression prevention (Cl, R)	i. Reframing depression (Cl, R)
	j. Preventing recurrent depression (Cl, R, S)
*Subtheme Barriers to an effective therapeutic space*	*Subtheme Barriers to an effective therapeutic space*
a. Emphasis on follow-up compliance (Cl, R)	a. Poor communication between the primary care clinician and other members of the care team (S, R)
b. Antidepressant side effects (Cl)	b. Depression
c. Over-reliance on antidepressant medication (Cl, R, S)	
d. Antidepressant non-adherence (Cl, R)	
e. Difficulties finding a good ‘fit’ with a counselor or psychotherapist (R, S)	
f. Short clinic visits and busy schedules (S)	

*C= competency; Cl= clinical; R= relational; S= system.

### Theme 1. patient’s journey to achieve depression care

i Facilitators (Clinician perspective)

Clinicians described the depression-related quality improvement projects at each clinic, additional staff training, and television advertisements for antidepressant medication as facilitating the journey to achieve depression care.

Clinician ‘a’, independent of the practice’s QI project, had trained staff to be compassionate with upset or irritable patients because these behaviors could be manifestations of depression. Clinician f felt patients’ references to television advertisements for antidepressant medications were a sign of decreased stigma related to both the disorder and its treatment. Several clinicians observed a trend toward patients more frequently listing of depression as a chief complaint than in past decades.

“So we’ve come a fair distance since then so I don’t have to spend quite so much time helping them understand what the diagnosis means and what the biologic basis may be and what treatment may be of some benefit and how long treatment needs to last in order to be effective and what is relapse and it’s much easier these days than it used to be.” [Clinician b]

ii Facilitators (Patient perspective)

While clinicians described systems-level facilitators, patients focused more on relational facilitators. A number of patients described supportive family members and a friendly office atmosphere as improving the journey to achieving depression care.

“And you know, the thing is about [clinician e’s] office is that everybody is always smiling and laughing and you know, how sometimes you go into places and there is a grumpy guy. … friendliness helps me to be able to say anything I feel I need to say, you know… Cause I’ve been to doctors where they are just intimidating to me. You know. And ok, it’s like let’s get this checked out and let’s get out of here.” [Patient 22]

iii Barriers (Clinician perspective)

Prominent barriers included lack of access to specialty mental health care, fear of opening “Pandora’s box” when considering a discussion around depression, challenges billing for depression counseling, and poor patient follow-up. Several clinicians noted that private psychiatrists were too often closed to new patients or were strictly fee-for-service. Clinician f, working at the federally qualified community health center, had telephone access to an on-call psychiatrist for consultation.

Manipulating billing codes, for example coding a symptom of depression such as “insomnia” instead of “depressive disorders,” to receive payment for treating depression was a barrier acknowledged by one clinician. Five clinicians lamented their newly diagnosed depressed patients’ poor follow-up rates following depression diagnosis.

“Somewhere between 30% and 50% of people do not come back for follow up. But those who are really depressed or really interested in making changes in their life, generally do come back.” [Clinician a]

iv Barriers to achieving care (patient perspective)

Again, clinicians focused on systems-level barriers, while patients generated a list of systems- and competency-level barriers. Patients concurred to an extent with clinicians that a fragmented health care system creates barriers to achieving care.

“So the cost to go to a psychiatrist is just ridiculous, and so I stayed within the bounds of what the primary care physician would do because I could get treated right away… It [depression care] has to be very easy to get to. You ask a depressed person to jump through too many hoops, big massive hoops. I’m not going to pay $250 a visit to see a psychiatrist. But not only financial hoops. It’s just a wall that, you know, is going to stop - would stop me from getting what I needed. They could make it easy for me, even if it was, “Okay, just give me one med to get me going. When I feel better and recover, I’ll take the longer route… Maybe the primary care facility could have a once-a-week psychiatrist.” [Patient 17]

In contrast to the clinicians, more patients seemed to perceive stigma as a persistent barrier.

“You know, yeah, I think there’s still, especially for men, there’s still a stigma associated with depression. I think that a lot of, you know – a lot of guys in general don’t necessarily know that they might be experiencing some depression and, therefore, don’t know what to do about it. Yeah, that’s the menace.” [Patient 17]

While most patients felt their current primary care clinician spent adequate time talking about depression with them, a number described moving on from previous clinicians for a range of different reasons.

“I did go on medication then and was on it for about 6 months and then went off of it… I think that was why I stopped because it wasn’t helping… But I was going to a different doctor then - one of those who rush you in and rush you out and this pill is going to cure everything. And it didn’t.” [Patient 29]

A few patients expressed concerns that primary care clinicians lacked expertise to treat depression.

“I don’t work with primary care on depression. I work with my therapist and my psychiatrist. I think the primary care physician would do well to just stay in the loop so they know what kind of medication this person’s and an overall view of how things are going.” [Patient 18]

Although some patients discussed their mental health diagnosis with front desk and medical assistant staff, one patient viewed the staff as potentially harmful to achieving depression care.

“For me… mental health has such a stigma that you know, you don’t want a lot of people to know about it. I’ve worked in a doctor’s office before … and truthfully I know the way people talk behind closed doors. … the least personal information that you really give them, I think the lower you fly on the radar and the better it is. You are there to talk to your doctor not to make friends.” [Patient 4]

### Theme 2: aiding patients to come to a personal understanding of depression

i Facilitators (Clinician perspective)

Several clinicians described educating patients about their newly diagnosed depression as very important. Three related the importance of trying to discriminate bipolar from unipolar depression.

ii Facilitators (Patient perspective)

The patient-level facilitators were primarily from the relational domain of care.

“So I would say, maybe, in 10^th^ grade, in high school, is when I realized I needed to get help for it [depression] because it runs every heavily in my mother’s side of the family. So I knew it was a genetic thing and I didn’t want to have this, but I kind of was like, ‘Okay’. I need to do something just to get— I got pretty bummed out and it was hard making choices and just going through every day and then I saw that [antidepressant] helping…” [Patient 20]

Psychotherapy was reported to instill enduring insights.

“I started with a psychiatrist who was more of an analyst kind of guy. And that’s not what I needed… So then I switched to a psychologist… and she was wonderful… she said you should put more trust in your own judgment. And then she brought me to the point where I realized some of my depression that I turned to anger towards me was because I didn’t want to be mad at my mom… she just gave me some good insights in a very loving and kind way. [Patient 23]

iii Barriers (Clinician perspective)

Two clinicians described somatic presentations of emotional distress as barriers to enabling patients to accept a depression diagnosis. One described disparate goals for the visit as a barrier, and provided the example of an otherwise healthy 24 year-old patient who was trying to qualify for disability. One clinician who had a subspecialization in substance abuse gave the opinion that 30-60% of depressed patients will have comorbid physical pain and/or substance abuse. One stated that when she is treating a patient for depression and detects possible substance or alcohol abuse that she will not continue treating the depression until the patient addresses the abuse problem.

iv Barriers (Patient perspective)

Patients nominated somatic expressions of emotional distress (2 patients) as a barrier.

*“*He was the one who initially diagnosed it and he did so after about four years of [me] hiding the possibility of having depression because I was very active. I just had these strange symptoms that just seemed disconnected and [clinician d] kept saying, well, you know, you can try this, or try that, but I really think you have depression.” [Patient 21]

### Theme 3: creating an effective therapeutic space

i. Facilitators (Clinician perspective)

Clinicians described the therapeutic space with a range of potential components (primarily clinical and/or relational domains of care).

Subtheme a – antidepressant medication

Clinician b referred the patient to psychiatry if a single antidepressant medication did not appear to be working. Clinician ‘a’ felt that an antidepressant was “almost necessary” if the patient is unable to change relationships, be more active, or get more sleep. Clinician c subspecialized in mental health care, and was proficient prescribing multiple concurrent psychotropic medications. At practice C the clinician started the antidepressant and often referred patients to a co-located psychiatric office for ongoing medication management.

Subtheme b – counseling/psychotherapy

All clinicians strongly advised counseling with or without antidepressant medication. Clinicians e and f observed a preference trend by gender.

“Many males will find it easier to take a pill than to go to counseling… But some are already in counseling or in those cases where I think it’s mild or they are not ready to go on medicine I’ll say well, have you thought about that?” [Clinician e]

Subtheme c- primary care clinician’s role

All primary care clinicians described multifaceted approaches to handling depression. Components included medication, counseling, physical activity, social support, diet, sleep hygiene, fish oil, vitamins, acupuncture, seeing a holistic practitioner, and other approaches. One described himself as directing ‘team effort’.

“If anything I’m the quarterback of this and I have some education with the biochemistry of this, but I don’t have … and I’ll say this to you – I don’t have the skill set to ask you about your mother and why your wife hates you and this and that… We need to get someone else in here as a team member to participate in this and you know, and sometimes that is very hard for people. And also you need to start exercising…And Patty is our new nutritionist.” [Clinician d]

Three clinicians required newly diagnosed patients to follow-up for a recheck within 1-3 weeks. Patients not complying were allowed a 1-month refill of antidepressant medication in order to reschedule the follow-up visit.

Empathetic listening was a relational domain of care described frequently by several clinicians as vital to meeting patient expectations. Clinicians describing themselves as empathetic listeners were also more likely to report falling behind often during their clinics.

“For me the biggest thing is that uh people feel like that they are listened to. I think that I find that a lot because people say that to me. ‘You really listened to me’, and I feel like people really want that. It’s really harder now because you have to see a lot of patients but I don’t know if you have to spend a whole lot of time, but I think you have to be there when you are there. [Clinician f]

Subthemes d and e – physical activity and social support

Clinician f described physical activity as one of 4 approaches he always recommended (also medication, therapy, and better nutrition), and several discussed ‘social support’ as helpful.

Subtheme f – depression prevention

Several clinicians discussed antidepressant medication to prevent recurrent depression. Clinician b raised the idea of providing antidepressant medication to susceptible persons at the advent of stressful situations or depressive symptoms and prior to descent into major depression. Clinician f noted that some of her patients develop skills that they use to prevent recurrence, although the specific skills were not listed.

ii Facilitators (Patient perspective)

Beyond the clinicians’ constrained and generic descriptions of the therapeutic space, in virtually every case patients described how they sought to bring together additional resources beyond their clinician’s recommendations that helped them in coping with depression. For both clinicians and patients the relational domain was most commonly described. While clinicians adhered closer to ‘evidence-based’ approaches (medication, counseling, physical activity), patients recruited a much broader range of elements, and seemingly unbeknownst to clinicians created unique personalized therapeutic spaces. Elements could be recruited at different phases of handling depression, from severe, prolonged bouts of depression, to handling a bad day, to preventing relapse.

Subtheme a - antidepressant medication

The role of antidepressant medication was complex. Patients often perceived medication as both a treatment and prevention strategy. Some patients perceived little need for other approaches to handling depression.

“I don’t know of anything beyond that because the Zoloft has worked very well for me and continues to. I’m just as happy as a little clam… I didn’t find the therapy of much use for me. The only thing that has really just worked fine is the Zoloft. [Patient 19]

More so than other elements of the therapeutic space, patients were very often ambivalent about taking antidepressant medication.

“I think a disadvantage of it [antidepressant medication] is you become used to it. Your body gets used to having that instead of, “Do you need more of it to keep working?” Or what if you eventually want to get off of it? Which I do. It’s been hard. I can’t really get off of it without relapsing type thing… So like throughout my life, I’ve been to the point I’ve been on it, I’ve gotten off of it, been on it, gotten off of it. It’s been back and forth. I know that I probably need to be more steady and stay on it or find a different way to deal with it.” [Patient 20]

Many patients were on long-term antidepressant therapy and several referred to worsening relationships when trying to wean off antidepressant.

“She recommended Celexa which I’ve been on, seven, eight years now… I ended up having to make the decision to stay on my Celexa and not breastfeed. But it’s just – when I came off, I was so miserable and so emotional. It was not good for me. It was not good for my family;” [Patient 11]

Subtheme b – counseling/psychotherapy

Some patients provided windows into the beneficial mechanisms of psychotherapy.

“Well, first I was working to see if I could figure out why I have such a social issue. Was there something I didn’t understand and I found out it appears to be all about self-esteem and body image when I am around people. And then we started dealing with working on controlling my cognitive reaction to my external environments. Like I can’t change the situation, but I can control my reactions to the situation. And a lot of exercises on how to manage my self-esteem and deal with working through the anxiety instead of fighting it”. [Patient 10]

Subtheme c- primary care clinician

As noted previously, patients were very appreciative of clinicians who spent time listening to and discussing their issues. Some patients noted the clinician’s role in prescribing antidepressant medication and referring to therapy, and one [patient 21] confirmed the more complex ‘multifaceted’ approach to depression that her clinician [clinician d] described herself as taking. Patients also valued those clinicians whom they perceived as highly skilled in treating depression.

“The specific doctor [clinician b] that I see said, ‘Hey, you know, [clinician c] is really up on this kind of stuff and you might really want to talk to her.’ I did and I really found that she is way more in tune with depression and, basically, what she call the serotonin imbalance… she has apparently studied and reads up on it and just seems way more knowledgeable about what’s going on in the treatment in that area.” [Patient 14]

Subtheme d – therapeutic aspects of work

While just one clinician commented briefly on work, in reference to a young, physically healthy patient asking for disability due to depression, several patients described *working* as a way to stay positive or to alleviate mental distress.

“Yeah. Cause if I do have anxiety it’s a good stress. Cause I’m on like… it’s almost like I’m on a deadline every minute… you’re just working. You are doing your shift. You are not messing around with each other. And for me that was like good stress…” [Patient 25]

Patient subthemes e-k were either not mentioned by clinicians or were only mentioned in passing.

Subtheme e - values

Personal *values* may be important for some patients when handling depression. One patient framed physical health not only as a personal value but as a potential influence on depression.

“And I mean… I’ve even went out and bought the South Beach Diet book and I’m going to be on the South Beach diet to keep my heart strong and be around for my grandchildren… and having been a product of depression my whole life and seeing what it did for my mother. She didn’t even want a stent put in her arteries. She just said let me die… It’s a debilitating disease. And that’s why I’m not ashamed of it. If I broke my leg I’d go get it set”. [Patient 22]

Subtheme f – family and nature

A number of patients described the critical role of *family members* in helping diminish depressive symptoms, and a handful described interactions with *nature* as part of the therapeutic space.

“My family had an intervention, basically, and said you need to go get on medication because you are driving yourself crazy and the rest of us. Not doing any good for anybody. So I said Ok. An I went to the doctor and got the pills…. How can you be sad or depressed or anything when you get on a horse and you go riding around and you just take in all the nature and you know, how can you have a bad day? If you have a bad day go for a ride. It’s totally different. It just changes you.” [Patient 26]

Subtheme g - faith

Faith is another factor that a handful of patients described as important to living with stress and depression.

“So I think faith is vital. My depression… I think everything just kind of… to me it all starts there. My religion, my faith has to be my base and the core of everything I am. It is something that keeps me grounded and keeps me hanging on when I just feel like running off and screaming or something.” [Patient 21]

Subtheme h – positive memories

A patient with prolonged grief after a friend died focused on *positive memories* of the deceased as both therapeutic and helpful to others.

“Well, he [clinician e] listens to me and he thinks I know myself well enough. I had dealt with it and put things in to a place in my mind where I could deal with it and treasure the memories and focus on the positive, the good memories. I needed to be around positive people and myself be positive and not wallow in this… it is a therapy for myself… I was talking to somebody I admired and felt close to and I said oh, you know, this was a part of [friend’s name]. I try to keep a part of her around me all the time.” [Patient 27]

Subtheme i - reframing depression experience

One patient described how her own approach to work took on new meaning as a result of having experienced depression.

“I work with a lot of elderly folks…. They just take whatever pills the nurse brings in a cup and they take so many they wouldn’t know if there was one being missed. And so… I try to look out… as you can imagine I’m very attuned to people’s needs in this area [depression] when I’m involved as a social worker…” [Patient 7]

Subtheme j – preventing recurrent depression

A recurring theme was the importance placed on listening, empathy, and striving to understand the patient’s experiences and preferences. Patients perceived long-term antidepressant use as helpful to prevent depression relapse. Interviewees used the primary care clinician as a way to prevent recurrent depression, for instance going to the doctor’s office when they felt their mood begin to worsen.

“…sometimes my depression is good. If I can tell that I’m like not feeling very good, at least now I’m able to let other people know, especially my doctor, what’s going on… one of the biggest things is that I felt that she truly did care. [Patient 15]

iii Barriers to an effective therapeutic space (clinician perspective)

Although expecting compliance to a guideline-based treatment protocol may improve outcomes for some patients, it may worsen outcomes for others, e.g. non-adherent patients who have access to receiving their antidepressant medication terminated. Two clinicians raised the issue of antidepressant side effects and non-adherence as barriers to achieving better outcome. Patients’ need to shop for a counselor or therapist with the right ‘fit’ was raised as an issue by 2 clinicians. Echoing patient concerns from theme 1, clinicians cited short clinic visits and busy schedules as limitations to an effective therapeutic space.

iv Barriers to an effective therapeutic space (patient perspective)

When asked about communication between care team members, several patients noted that meaningful communication between members of the care team was uncommon. One patient took it upon himself to keep each clinician informed. A few patients noted that severe depression could be so overwhelming that they would be unable to activate or engage in aspects of the therapeutic space.

## Discussion

This qualitative study is among the first to simultaneously explore and compare primary care patients’ and their primary care clinicians’ perceptions of facilitators and barriers to achieving care for depression. Patients and clinicians provided partially overlapping and complementary descriptions of facilitators and barriers to patients’ abilities to achieve the 3 emergent themes, receiving depression care, coming to a personal understanding of their depression, and constructing a therapeutic space. These data underscore the complexity of measuring and understanding the patients’ experiences of receiving care for an often chronic condition, depression. Results from this study may inform ongoing efforts to define measures [33] of the patient experience, and may contribute to modifications to improve the patient’s experience within evolving primary care systems such as PCMH in the United States.

### Theme 1: journey to achieving depression care

Clinicians and patients from different practices nominated inter-related factors, a training for office staff to provide better customer service and a friendly office atmosphere, respectively, as key to facilitating depression care. While some QI projects may improve patient retention at small practices, [34] future research would investigate how critical staff friendliness is to retaining patients. It may be difficult to achieve highly confidential mental health care at primary care clinics as currently organized. Access to fragmented mental health care sites may enable those patients preferring more privacy around their mental health issues to receive satisfactory highly confidential care [35].

We uncovered a tension between patients who were dissatisfied with clinicians who appeared hurried and uncaring, and clinicians who worried that discussing depression could open “Pandora’s box” of lengthy discussions around depression and set them irrecoverably behind in their clinic schedules. These findings echo those of Palmer et al. who found that “listen, understand and empathize” was the most often cited depression-related task that depressed patients thought clinicians should provide [26].

In an international study, patients and stakeholders agreed health care systems should fund longer clinic visits [26]. Supporting this concept, the “group health medical home” in the United States conducted a whole-practice transformation including increased clinic visit durations from 20 to 30 minutes. Early findings include improved patient satisfaction, cost savings, and less physician burnout [36]. However, with more patients receiving insurance in the U.S. health care system, and limited primary care providers, it appears unlikely that visit times would expand in the foreseeable future. Studies investigating possible links between time spent discussing depression during the primary care visit and better clinical outcomes might support longer primary care visits.

Finally, the contrast between clinician opinions that stigma has lessened over previous decades and patient reports of ongoing stigma suggests that clinicians may sometimes underestimate the negative of impact of stigma on achieving depression care. Further study of a potential mismatch between clinicians’ and their patients’ assessments of stigma may be warranted.

### Theme 2: personal understanding of depression

Clinicians and patients agreed on a clinical barrier, somatic manifestations of mental distress, to helping patients come to a personal understanding of their depression. Depressed patients’ exclusively somatic chief complaints were associated with worse clinical outcomes in a collaborative care trial, [37] and further study of somatic presentations of depression has been recommended [25]. Two scenarios in which different clinicians felt blocked helping patients understand their depression were: a. patients suspected of trying to inappropriately obtain disability for depression; and b. probable concurrent substance abuse disorder. How to best respond when presented with these dilemmas may provide foci for future investigation.

### Theme 3: therapeutic space

Clinicians displayed a surface understanding of the patients’ therapeutic spaces, most often describing guideline-based components such as therapy, medication, and physical activity, and possibly nutrition, social support, and ‘holistic’ therapies. Clinicians recognized depression as an often chronic or recurrent disorder, [19] yet conceived its treatment based most often on episodic short-term approaches.

For the patient, the ‘therapeutic space’ constituted a versatile array of important caregivers, family members, medications and other therapeutic products or treatments, inner qualities such as faith, positivism, values and memory, and outward connections to workplace, family role, physical activity, and nature. Patients combined personal and ‘outside the box’ approaches with professional (antidepressant medication, therapy) approaches in their support webs, contrasting with a recent finding that depressed patients preferred personal over professional approaches [38].

Our description of the therapeutic functional space builds on the work of Cooper et al. and McMullen and Stoppard, who have noted that factors such as spirituality, social support systems, coping strategies, life experiences, and patient-provider relationships contribute to mental well-being [39,40]. While the “journey to achieve depression care” overlaps to a large extent with Kovandzic’s “space of access to primary mental health care” [35], “therapeutic space” overlaps with and builds more upon Dowrick et als. descriptions of ‘ordinary magic’ (drawing on existing social supports and affectional bonds) and personal medicine (building on personal strengths and expanding positive emotions) [38]. Future research would determine how to ‘measure’ the therapeutic space with quantitative instruments.

The components of the therapeutic space may be considered from a sociological perspective as appropriate responses to the depressive influences of 3 cultural trends, the medicalization of suffering, disconnection from work and loving relationships, and ‘postmodern existence’ [20]. The therapeutic space may serve to push back against these de-humanizing trends by humanizing suffering, creating meaningful connections, and embracing nurturing aspects of pre-modern existence. Patients described the space as a way to handle not only current depression but to try and avert future depression, and acknowledged severe depression’s ability to negatively impact or ‘shrink’ the therapeutic space. About 1 in 3 patients with an index depressive episode experiences a second episode within 1 year, [41] and almost 90% relapse within 15 years [42]. Because most depressed patients will relapse, research would investigate whether encouraging patients with an initial depressive episode to begin developing as substantial a therapeutic space as possible wards off future depressive episodes.

Another relevant research focus would be to explore the extent that depressed persons draw on pre-existing supports and bonds versus creating new supports, bonds, and other connections, and how therapeutic space may affect the environment around the depressed person. A re-connective therapeutic space might be considered as a feedback loop to slow down the effects of the de-humanizing processes described above, while a creative therapeutic space might be considered as rolling back the processes or even generating novel humanizing relationships. A randomized trial would investigate whether the primary care clinician might help patients recover from depression by learning about, supporting and strengthening each depressed patient’s unique therapeutic space.

### Limitations

The study is limited in that respondents were patients from purposefully selected small to medium sized primary care clinics, and as such represent the perspectives of persons with a depression diagnosis from that population. Most of the patients reported multiple episodes of depression, consistent with observations that depression in primary care is most often chronic or recurrent in nature*.* However, more complex patients with chronic depression may have been more likely to respond to the invitation than patients with history of mild or brief depression. Possible participants were identified for history of depression treatment by ICD-9 code, an approach with sensitivity and specificity over 70% for history of major depression [43].

Patients were mostly middle- to upper-class, insured, English-speaking, and attending primary care practices, and therefore do not represent a completely representative sample of US citizens with depression. However, the majority of diagnosed depression cases are treated at least partly in primary care practices [44]. While about 30% of depressed patients are typically male, men are under-represented in our study, comprising only 16.7% of the sample (5 subjects), thereby limiting ability to generalize results to males. We believe the findings reported herein are generalizable to a range of primary care adults with history of depression as the emergent themes were consistent across sites. Another strength is that the theoretical perspectives of the research team were mixed (e.g. cultural anthropology, public health, medicine).

## Conclusion

Our description of the journey to achieve depression care, coming to a personal understanding of depression, and the creation of the therapeutic space begins to address previous recommendations to study the patient experience in order to inform how best to restructure primary care to better meet the clinicians’ and patients’ preferences and needs, thereby improving the patient’s experience [45]. Developing models of care that optimize the patient’s experience, while also addressing the patients’ expectations and preferences regarding medical care, may be as or more important than the current emphasis on developing infrastructure and information technology at the PCMH. Qualitative data describing patients’ and clinicians’ perceptions of achieving care for depression may help inform development of both an improved primary care environment and instruments that effectively measure patients’ notions of a person-centered experience [9].

This research article adheres to the RATS guidelines on qualitative research.

Qualitative research review guidelines – RATS.

http://www.biomedcentral.com/authors/rats accessed 12/31/13

## Competing interests

The authors declare that they have no competing interests.

## Authors’ contributions

RK drafted the manuscript. DW and PN conceived the study. RK, DW, BT, and PN designed the methods. RK collected the data. RK, DW, BT, and PN performed the qualitative content analysis. RK, DW, BT, and PN helped to draft the manuscript. All authors have read and approved the final manuscript.

## Pre-publication history

The pre-publication history for this paper can be accessed here:

http://www.biomedcentral.com/1471-2296/15/13/prepub

## Supplementary Material

Additional file 1 Overview of qualitative patient interview – original v1. Click here for file

Additional file 2 Patient interview guide– original version 1. Click here for file
