# Supplementary material for: A qualitative comparison of primary care clinicians’ and their patients’ perspectives on achieving depression care: implications for improving outcomes
Source: BMC Fam Pract. 2014 Jan 15;15:13. doi: 10.1186/1471-2296-15-13 (PMC3907132; doi:10.1186/1471-2296-15-13)
Supplement: Additional file 1 — Overview of qualitative patient interview – original v1. [file 1471-2296-15-13-S1.doc]

**OVERVIEW OF QUALITATIVE PATIENT INTERVIEW – Original V1**

**Stems and Specific Related Questions. Clinician**

1. What does depression mean to the physician?

a. Based upon clinical and/ or personal experience or training, how would you describe depression?

b. On what training or experience do you base your description?

c. In your opinion, what are the causes of depression?

d. How does depression relate to overall health?

e. Do you think that depression related to other chronic physical (or mental) conditions? If so, how?

f. How does depression relate to social circumstances (e.g. homelessness, joblessness, traumatic life events, history of interpersonal or other violence) ?

1. What are the physician’s perceptions of ways to treat depression? What are his or her expectations of the course of depression?
   1. What are some ways to help patients alleviate depressive symptoms?
   2. How do you view your role in treating depression in your patient population? Can you give one or two examples?
      1. Is the primary care doctor an initial contact for providing referral, a person with whom to obtain counseling and/ or medical treatment, or both?
      2. What do you see as your strengths and weaknesses regarding helping your patient recover from depression?

Ii. What are challenges or barriers you experience when trying to treat depression?

- 1. Is the physician essential to the care-giving process for depression, or can others in the practice be viewed as caregivers?
  2. What are the perceived advantages and disadvantages of different treatment modalities?
     1. Which treatment modalities are acceptable? (specialty counseling (group, individual), medication, treatment with medication and therapy, watchful waiting)
     2. How acceptable or not acceptable are they to you?
     3. How do you most often treat your depressed patients?
  3. What is the most typical course of depression you see in your practice? (e.g. waxing and waning, single event, rare and short-lived, related to stressful events etc.)

**.**
