# Supplementary material for: A qualitative comparison of primary care clinicians’ and their patients’ perspectives on achieving depression care: implications for improving outcomes
Source: BMC Fam Pract. 2014 Jan 15;15:13. doi: 10.1186/1471-2296-15-13 (PMC3907132; doi:10.1186/1471-2296-15-13)
Supplement: Additional file 2 — Patient interview guide– original version 1. [file 1471-2296-15-13-S2.doc]

**PATIENT INTERVIEW Guide– Original Version 1**

**Stems and Specific Related Questions. Patient.**

1. What are the patient’s perceptions of ways to treat depression? What are his or her expectations of the course of depression?

- 1. What are some ways to help alleviate depressive symptoms?
  2. When you or someone you know has depression, is your primary care doctor someone you talk with about depression? Why?
     1. Is the primary care doctor an initial contact for receiving referral, a person with whom to obtain counseling and treatment, both?
     2. What are strengths and weaknesses of the doctor regarding treatment of depression?
     3. What are weaknesses or barriers to treating depression?

1. Is the physician essential to the care-giving process for depression, or can others in the practice be viewed as caregivers?
2. What are the perceived advantages and disadvantages of different treatment modalities?
   - 1. Which treatment modalities are acceptable? (specialty counseling (group, individual), medication, treatment with medication and therapy, watchful waiting)
     2. How acceptable or not acceptable are they?
3. What has been the course of depression over your life? (e.g. waxing and waning, single event, rare and short-lived, related to stressful events etc.)

V2

**PATIENT INTERVIEW Guide–Version 2** (incorporates questions pertaining to themes emerging from earlier patient interviews)

**Stems and Specific Related Questions. Patient.**

1. Tell me about your depression.

a. Thinking back to the events of depression over your life, can you tell me about the experiences?

b. Describe what, if anything, was causing you to feel that way?

i. How were the good times different from the bad times?

ii. How did your interactions with health care providers, counselors or other persons regarding depression evolve or change over time?

iii. What kinds of things have you done specifically to try and prevent the relapse toward major depression?

iv. How have others, including your primary care provider, helped?

1. How was depression originally addressed at the practice?
   - 1. What are specific approaches you take to recover from depression?
     2. If your health care providers are aware of these approaches, can you tell me how they reacted?
     3. If your health care providers are NOT aware, how do you imagine they might react and why?
2. What was it that finally got you to get help with depression?
   - 1. How aggressive should the doc be in identifying depression?
     2. How involved/ active would you like the doctor to be in managing depression?
3. When you or someone you know has depression, is your primary care doctor someone you talk with about depression? Why?
   - 1. Is the primary care doctor an initial contact for receiving referral, a person with whom to obtain counseling and treatment, both?
     2. What are strengths and weaknesses of the doctor regarding treatment of depression?
     3. What are weaknesses or barriers to treating depression?

2. Is the physician essential to the care-giving process for depression, or can others in the practice be viewed as caregivers?

a. What are the perceived advantages and disadvantages of different treatment modalities?

i. What do you see as the goal of depression treatment?

- 1. Which treatment modalities are acceptable? (specialty counseling (group, individual), medication, treatment with medication and therapy, watchful waiting)
     1. How acceptable or not acceptable are they?
